# Antimicrobial Resistance Glides in the Sky—Free-Living Birds as a Reservoir of Resistant *Escherichia coli* With Zoonotic Potential

**DOI:** 10.3389/fmicb.2021.656223

**Published:** 2021-04-09

**Authors:** Magdalena Skarżyńska, Magdalena Zaja̧c, Arkadiusz Bomba, Łukasz Bocian, Wojciech Kozdruń, Marcin Polak, Jarosław Wia̧cek, Dariusz Wasyl

**Affiliations:** ^1^Department of Microbiology, National Veterinary Research Institute, Puławy, Poland; ^2^Department of Omics Analyses, National Veterinary Research Institute, Puławy, Poland; ^3^Department of Epidemiology and Risk Assessment, National Veterinary Research Institute, Puławy, Poland; ^4^Department of Poultry Diseases, National Veterinary Research Institute, Puławy, Poland; ^5^Department of Zoology and Nature Protection, Institute of Biological Sciences, Maria Curie-Skłodowska University, Lublin, Poland

**Keywords:** antimicrobial resistance, free-living birds, AMR, wildlife, *E. coli*

## Abstract

Antimicrobial resistance (AMR) is one of the most important global health concerns; therefore, the identification of AMR reservoirs and vectors is essential. Attention should be paid to the recognition of potential hazards associated with wildlife as this field still seems to be incompletely explored. In this context, the role of free-living birds as AMR carriers is noteworthy. Therefore, we applied methods used in AMR monitoring, supplemented by colistin resistance screening, to investigate the AMR status of *Escherichia coli* from free-living birds coming from natural habitats and rescue centers. Whole-genome sequencing (WGS) of strains enabled to determine resistance mechanisms and investigate their epidemiological relationships and virulence potential. As far as we know, this study is one of the few that applied WGS of that number (*n* = 71) of strains coming from a wild avian reservoir. The primary concerns arising from our study relate to resistance and its determinants toward antimicrobial classes of the highest priority for the treatment of critical infections in people, e.g., cephalosporins, quinolones, polymyxins, and aminoglycosides, as well as fosfomycin. Among the numerous determinants, *bla*_CTX–M–15_, *bla*_CMY–2_, *bla*_SHV–12_, *bla*_TEM–1B_, *qnrS1*, *qnrB19*, *mcr-1*, *fosA7*, *aac(3)-IIa*, *ant(3”)-Ia*, and *aph(6)-Id* and chromosomal *gyrA*, *parC*, and *parE* mutations were identified. Fifty-two sequence types (STs) noted among 71 *E. coli* included the global lineages ST131, ST10, and ST224 as well as the three novel STs 11104, 11105, and 11194. Numerous virulence factors were noted with the prevailing *terC*, *gad*, *ompT*, *iss*, *traT*, *lpfA*, and *sitA*. Single *E. coli* was Shiga toxin-producing. Our study shows that the clonal spread of *E. coli* lineages of public and animal health relevance is a serious avian-associated hazard.

## Introduction

The scale of bacterial resistance to antimicrobials is one of the most important global health concerns. Although antimicrobial resistance (AMR) is an ancient and natural phenomenon, the widespread use of antimicrobials in human and veterinary medicine and also in agriculture contributes to its pandemic dissemination (D’Costa et al., 2011; Martínez, 2012; World Health Organization [WHO], 2014). The enormous consequences of AMR cover most of all treatment failures and increased mortality. However, economic losses as a consequence of AMR increase are also significant and they generate extra healthcare costs and a decrease of productivity (European Centre for Disease Prevention and Control, 2009). Nevertheless, losses in agriculture and animal production sector should also be listed here.

The use of antimicrobials (AMU) is considered one of the main drivers of AMR emergence; therefore, the impact of drug residues in municipal waste waters and of organic fertilizers in agriculture is indisputable (Swift et al., 2019). Several other environmental factors may affect AMR dissemination. Chemicals like disinfectants, fungicides, and pesticides widely used in agriculture may effectively co-select AMR (Hansen et al., 2007; European Commission, 2009; Hobman and Crossman, 2015). Similarly, heavy metals (e.g., copper) used in antifungal plant protection agents should also be deliberated (Borkow and Gabbay, 2005; Hobman and Crossman, 2015). All those pollutants contribute to the selection and spread of AMR in the environment affecting wildlife.

Consequently, free-living animals may serve as a reservoir of AMR determinants. The scale of AMR in wildlife, despite several reports on this subject, continually seems to be incompletely known and underestimated (Guenther et al., 2010a; Arnold et al., 2016; Mo et al., 2018; Wasyl et al., 2018). However, in the assessment of wildlife input to the AMR spread, different animal species with diverse habitats and feeding behaviors need to be considered. It should be emphasized here that migratory species seem to pose a significant threat to AMR dissemination. Seasonal migration, often over long distances and between continents, contributes to greater exposure of animals and their intestinal microbiome to various AMR drivers (Arnold et al., 2016). In this context, the role of free-living birds as AMR carriers is noteworthy and has already drawn the attention of several researchers (Pinto et al., 2010; Literak et al., 2012; Poirel et al., 2012; Veldman et al., 2013; Vredenburg et al., 2014; Oh et al., 2016; Ahlstrom et al., 2018; Zurfluh et al., 2019). To illustrate a ubiquitous and diverse avian community, it covers species that avoid human proximity, such as the golden oriole (*Oriolus oriolus*), as well as species living close to human settlements, like the house martin (*Delichon urbicum*). Other examples of the puzzle are herring gulls (*Larus argentatus*) known to prey on landfills and around sewage treatment plants, areas polluted with a variety of AMR determinants and its affecting factors (Bonnedahl and Järhult, 2014), and white stork (*Ciconia ciconia*) popular in Polish spring and summer landscape and wintering far away in Africa.

Recent developments in sequencing techniques provide an invaluable tool to elucidate the background and pathways of AMR transmission. Accurate genotypic characterization of bacteria enables to examine the genotypes circulating among environments and to identify their possible links to clinically relevant resistant pathogens and, thus, human and animal infections. Besides a somewhat unrealistic direct contact with free-living birds, there is a serious possibility of livestock or human contact with, for example, bird droppings (Dolejska and Literak, 2019). A flash point for the current study was multidrug-resistant (MDR) *Escherichia coli* derived from an individual of green woodpecker (*Picus viridis*) found in nature. The strain was isolated in 2013 from fresh feces collected during the delivery of birds to a veterinary clinic due to clinical symptoms.

In this study, we applied microbiological culture methods commonly used in the official monitoring of slaughter animals (European Food Safety Authority, 2019) to investigate the AMR status of *E. coli* isolated from free-living birds. The samples were derived from birds coming from natural habitats and rescue centers. The application of whole-genome sequencing (WGS) aimed to characterize AMR determinants and plasmids associated with AMR horizontal transfer and also to investigate the epidemiological relationships and virulence potential of *E. coli* isolated from free-living birds.

## Materials and Methods

### Sample Collection

A total of 69 samples (20 intestines, 44 feces, 4 goiter swabs, and 1 stomach sample) from 68 free-living birds were collected between 2017 and 2020 within a convenience sampling in the National Reference Laboratory for Antimicrobial Resistance (NRL) at the National Veterinary Research Institute (NVRI) in Puławy, Poland. The most prevalent tested group constituted birds of prey that belonged to Accipitriformes (*n* = 23) and Strigiformes (*n* = 3). The other group consisted of migratory species of Pelecaniformes (*n* = 19) and ubiquitous Passeriformes (*n* = 15) representing the most abundant avian order. The dataset was completed with orders represented by Anseriformes (*n* = 5), Gruiformes (*n* = 1), Charadriiformes (*n* = 1), and Columbiformes (*n* = 1).

Twenty-two samples were derived from deceased birds, mostly birds of prey (*n* = 9) and waterbirds (*n* = 6), and sent to the NVRI for diagnostics purposes (e.g., defining the cause of death). The second subset of samples was collected from 15 white storks residing in the Center for Rehabilitation of Free-living Birds in Bukwałd due to mechanical injuries. Antimicrobial treatment status of this group remained unknown. Eight fresh feces samples (six from birds of prey and two from white storks) were collected from animals at the Bird of Prey Rehabilitation Center in Da̧brówka on the day of release. The birds were not treated with antimicrobials during their stay in the rescue center. The remaining samples (*n* = 24) were taken from animals in their natural environment during the ringing activity or nest inspection carried out in 2019 in Lublin region (S-E Poland) by ornithologists from the Department of Zoology and Nature Protection, UMCS. The study fulfilled the current Polish law and was permitted by the Ministry of the Environment (approval number: DL-III.6713.11.2018.ABR) and the General Directorate for Environmental Protection (approval number: DZP-WG.6401.03.2.2018.jro). The Regional Directorate for Environmental Protection (RDOŚ) in Lublin allowed for the research project through a letter (approval number: WPN. 6401.6.2018.MPR). Ten samples of birds sampled in nature came from juvenile birds of prey—marsh harrier (*Circus aeruginosus*). All tested bird species, their origin, and included sample types are presented in Supplementary Table 1.

### Isolation and Identification of *E. coli*

Usually, on the day following their collection, the samples were cultured on buffered peptone water for 18 ± 3 h at 37°C and then streaked on MacConkey agar (Oxoid, Hampshire, United Kingdom), MacConkey agar supplemented with cefotaxime (1 mg/L, Oxoid, Hampshire, United Kingdom), chromID^TM^ CARBA, chromID^TM^ OXA-48 agar (bioMérieux, Marcy l’Etoile, France), and MacConkey supplemented with colistin (2 mg/L, Oxoid, Hampshire, United Kingdom) for isolation of commensal, cephalosporin-, carbapenem-, and colistin-resistant *E. coli*, respectively. Suspected colonies were identified with matrix-assisted laser desorption/ionization time-of-flight mass spectrometry (MALDI-TOF MS, Microflex LT MALDI Biotyper; Bruker Biosciences, Billerica, MA, United States).

### Antimicrobial Resistance Testing

All *E. coli* were tested for antimicrobial susceptibility with the microbroth dilution method (Sensititre; TREK Diagnostic Systems, Thermo Fisher Scientific, Waltham, MA, United States). Resistance tests to nine antimicrobial classes—beta-lactams, quinolones, phenicols, aminoglycosides, folate path inhibitors, tetracyclines, polymyxins, macrolides, and glycylcyclines—were performed with EUVSEC plates (as described in Table 1 of the Annex to 2013/652/EC). For all isolates resistant to cephalosporins, the second panel (EUVSEC2 according to Table 4 of the Annex to 2013/652/EC) was applied. The European Committee on Antimicrobial Susceptibility Testing (EUCAST) epidemiological cutoff values (ECOFFs) for minimum inhibitory concentration (MICs) were used as interpretation criteria. *E. coli* strain was regarded as resistant (non-wild type, NWT) when MIC values above the cutoff were obtained. Among the NWT category, strains resistant to at least three antimicrobial classes were referred as multidrug-resistant (MDR) (Magiorakos et al., 2012). *E. coli* with all MIC values below the ECOFF were recognized as susceptible (wild type, WT). The procedure mirrored the official AMR monitoring implemented in the EU according to Directive No. 2013/652/EC.

### Whole-Genome Sequencing

Seventy-one *E. coli* strains, including an archival strain from a woodpecker, have been subjected to whole-genome sequencing. Extraction of DNA was prepared with Maxwell^®^ RSC Cultured Cells DNA Kit—Automated DNA Purification from Mammalian and Bacterial Cultured Cells (AS1620 Promega, Madison, Wisconsin, United States) according to the manufacturer’s instruction with Maxwell^®^ RSC Instrument (Promega, Madison, Wisconsin, United States). For yield and purity check, all samples were measured with NanoDrop^TM^ One following extraction (Thermo Scientific, Waltham, MA, United States). DNA libraries prepared with Library Preparation Kit (Illumina, Inc., San Diego, CA, United States) according to the manufacturer’s instructions were sequenced with the MiSeq platform (Illumina, Inc., San Diego, CA, United States). Paired-end sequencing per flow cell (2 × 300) was applied.

### Bioinformatic and Statistical Analyses

FastQC 0.11.5 was used for the raw reads quality check and Trimmomatic 0.36 (Bolger et al., 2014) for the read trimming. Corrected reads were *de novo* assembled by SPAdes 3.9.0 (Bankevich et al., 2012). Resistance and plasmid identification was conducted using abricate 1.0.1 (Seemann, 2019) against ResFinder (Zankari et al., 2012) and PlasmidFinder (Carattoli et al., 2014) databases (2020-07-25) with identity threshold 95% and selected minimum length 60%. PointFinder software 3.1.0 was applied for the identification of chromosomal point mutations (database: 2019-07-02) (Zankari et al., 2012). For the identification of multilocus sequence type (MLST, ST), we used the MLST 2.0 tool (Larsen et al., 2012) with database version 2.0.0 (2020-05-04). Virulence factors were analyzed with VirulenceFinder 2.0 and its database of 2020-05-29 with% ID threshold 90% and minimum length 60% (Joensen et al., 2014). Submission of *E. coli* with unknown ST to EnteroBase v1.1.2^1^ allowed to assign new sequence type using the Achtman 7 Gene MLST algorithm (Zhou et al., 2020).

CSI Phylogeny 1.4 (call SNPs and infer phylogeny) CGE with input parameters—minimum depth at single nucleotide polymorphism (SNP) positions: 10, relative depth at SNP positions: 10, minimum distance between SNPs (prune): 10, minimum SNP quality: 30, minimum read mapping quality: 25, minimum *Z*-score: 1.96—was applied for phylogeny tree preparation (Kaas et al., 2014). As reference genome of *E. coli* from one of the two most represented ST types was chosen (14P KOL). The online tool iTOL v5 was applied for phylogeny tree visualization (Letunic and Bork, 2019).

The sequences were deposited at the European Nucleotide Archive (ENA)^2^ under accession number PREJB42669^3^.

The variability of the noted MLST and virulence genes was measured with Simpson’s diversity index (Hunter and Gaston, 1988).

To determine the statistical difference in the occurrence of resistant *E. coli* between groups of birds, a chi-square test with the appropriate correction was applied (Supplementary Table 2). Resistance in different groups of birds was assessed with a 95% confidence interval.

## Results

### Phenotypic Results of Antimicrobial Resistance

A total of 73 *E. coli* were isolated. Sixty were obtained from MacConkey agar, six from MacConkey supplemented with cefotaxime, and seven from MacConkey with colistin. No carbapenem-resistant *E. coli* were found. To avoid duplicate testing of the same strain, three isolates were excluded, as they were obtained on different culture media from the same sample but showed identical minimum inhibitory concentration (MIC) values. As a result, 70 strains were included in the comparison of resistance in different bird groups. Half of the tested *E. coli* were found resistant (*n* = 35, 50.0%) and most of them (*n* = 27, 38.6%) were MDR (resistant to at least three antimicrobial classes). The analyses showed significant differences between the number of resistant strains isolated from birds sampled in nature and all the other groups (*p*-values 0.0047–0.0003) (Supplementary Table 2). Susceptible *E. coli* were derived mostly from birds sampled in nature. Yet, two strains derived from blue tit (*Cyanistes caeruleus*) sampled in nature exhibited non-wild-type MICs with 8 and 11 antimicrobials each.

**FIGURE 1:**
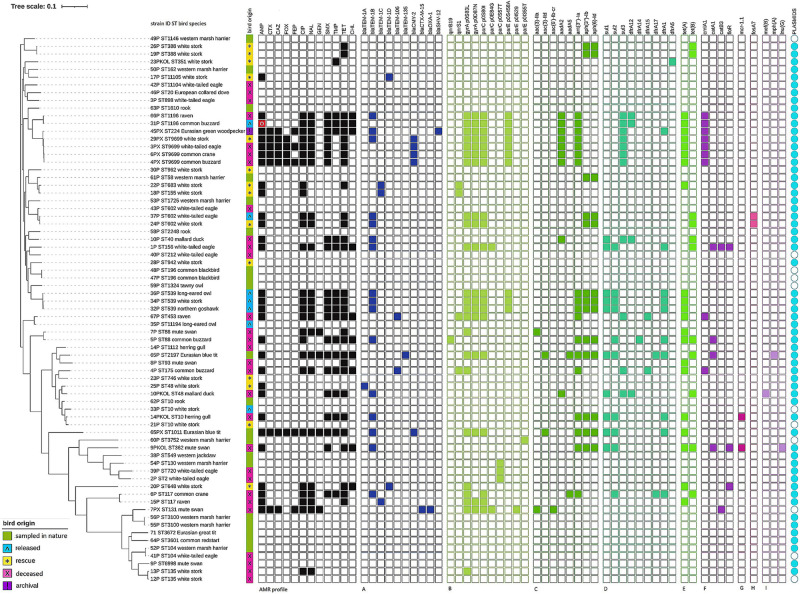
Phylogeny of *Escherichia coli* strains isolated from wild birds. Full and empty squares depict the presence or absence of AMR or AMR determinants. AMR profile in black—phenotypic AMR profiles confirmed by the presence of AMR determinants. Red square with white circle means phenotypical resistance not confirmed by genotypic testing. **(A)** Beta-lactam, **(B)** quinolone, **(C)** aminoglycosides, **(D)** folate path inhibitors, **(E)** tetracyclines, **(F)** phenicols, **(G)** colistin, **(H)** fosfomycin, **(I)** macrolide and lincosamide resistance determinants. Visualization prepared with the online tool iTOL v5.

Of all the antimicrobials assessed, ampicillin and tetracycline resistance dominated (41.4%, each) followed by quinolones (35.7 and 31.4% for ciprofloxacin and nalidixic acid, respectively), as well as folate path inhibitors (28.6% sulfamethoxazole and almost 22.9% trimethoprim). A lower percentage of AMR was observed for chloramphenicol (12.9%) and third-generation cephalosporins (8.6%). Four strains (5.7%) were resistant to gentamicin. Supplementary Table 3 presents detailed MIC value distribution of the tested *E. coli* on both applied panels. Overall, 20 different AMR profiles were noted (Figure 1).

### AMR Determinants and Plasmid Replicons

Cephalosporin resistance was defined as extended-spectrum beta-lactamases (ESBLs) and AmpC-type cephalosporinase production. ESBL determinants were identified as *bla*_SHV–12_ (archival woodpecker *E. coli*) and *bla*_CTX–M–15_ [*n* = 1, mute swan (*Cygnus olor*)—deceased]. AmpC-type cephalosporinases were determined by *bla*_CMY–2_ [*n* = 5, blue tit—nature, white-tailed eagle (*Haliaeetus albicilla*), buzzard (*Buteo buteo*), crane (*Grus grus*)—deceased, white stork—rescue]. Resistance to ampicillin was linked to *bla*_TEM–1B_ most often. In one ampicillin-resistant *E. coli* from buzzard (released), any relevant determinant was noted that could confer resistance for beta-lactams.

Mutations in quinolone resistance determining region (QRDR) dominated ciprofloxacin and nalidixic acid resistance, and *gyrA* substitution S83L was noted in the majority of 26 resistant strains (*n* = 23). Three quinolone-susceptible strains carried single *parC* mutations [*n* = 1, marsh harrier (*Circus aeruginosus*)—nature; *n* = 2, white-tailed eagle —deceased] and one wild-type strain possessed *parE* mutation [marsh harrier—nature].

Plasmid-mediated quinolone resistance (PMQR) genes were identified as *qnrS1* [*n* = 4; two white storks—rescue, raven (*Corvus corax*), and buzzard—deceased] and *qnrB19* (*n* = 1; buzzard—deceased). The gene *aac(6′)-Ib-cr* that determines AMR toward quinolones and aminoglycosides was noted in a strain from deceased mute swan carrying *bla*_CTX–M–15_ and *bla*_OXA–1_.

The genes *aac(3)-IIa* (*n* = 2, mute swan—deceased) and *aac(3)-IId* (*n* = 2, blue tit—nature) were associated with gentamicin resistance. Of all determinants noted, genes encoding resistance toward aminoglycosides other than gentamicin (e.g., streptomycin not tested phenotypically) were the most abundant (*n* = 55), and among them, *ant(3″)-Ia* (*n* = 18), *aph(3″)-Ib* (*n* = 13), and *aph(6)-Id* (*n* = 13) prevailed. Genes *sul1*, *sul2*, *sul3*, and *dfrA1* were the most often noted among strains resistant to folate path inhibitors, while genes *tet*(A) (*n* = 21) and *tet*(B) (*n* = 11) dominated in tetracycline-resistant *E. coli*. Resistance toward phenicols was determined by *cmlA1*, *catA1*, *catB3*, and *floR*. Genes *cmlA1* and *catB3* were also found in five strains that were chloramphenicol susceptible.

The *mcr-1* gene conferring colistin resistance was noted in two strains with MIC for this antimicrobial below the cutoff (MIC = 2 mg/L). Both *E. coli* were found in deceased water birds (herring gull and mute swan). WGS also revealed the presence of *fosA7* determining resistance toward fosfomycin (not tested phenotypically) in two *E. coli* (white stork—rescue and white-tailed eagle—released). Three genes that confer resistance for macrolides [*mef*(B), *mph*(A)] and lincosamides [*lnu*(G)] were noted as well (Figure 1).

All genotype–phenotype discrepancies in case of *mcr-1* finding in colistin wild-type strain as well as *E. coli* susceptible to chloramphenicol that carried *cmlA1* and *catB3* were confirmed by repeated susceptibility testing. Resistance to ampicillin in *E. coli* without any relevant AMR gene was also verified.

Multiple plasmid replicons (*n* = 30) were found dispersed in most of the tested *E. coli* (*n* = 60). The most frequent replicon was IncFIB(AP001918) (*n* = 47). Few other replicons IncI1_1_Alpha, p0111, IncFIC(FII), IncQ1, and IncFII occurred in a similar number of strains (from 8 up to 12 strains) (Supplementary Figure 1). Ten from 11 plasmid replicon-free strains were pan-susceptible and AMR gene-free (Figure 1). The exception was one strain from deceased mute swan carrying five resistance genes including genes *bla*_CTX–M–15_, *bla*_OXA–1_, and *aac(6’)-Ib-cr*. Chromosomal location of antimicrobial resistance genes in that strain was confirmed with MinION long-read sequencing (Oxford Nanopore Technologies, data not presented).

Seventeen strains possessed one plasmid replicon and three of them carried resistance genes. Among 34 *E. coli* with two up to four identified plasmid replicons, 25 strains were recognized as non-wild type (NWT), including 17 MDR. Nine *E. coli* carried from five up to eight plasmid replicons and all were MDR (Figure 1 and Supplementary Figure 1).

### Virulence Genes

**FIGURE 2:**
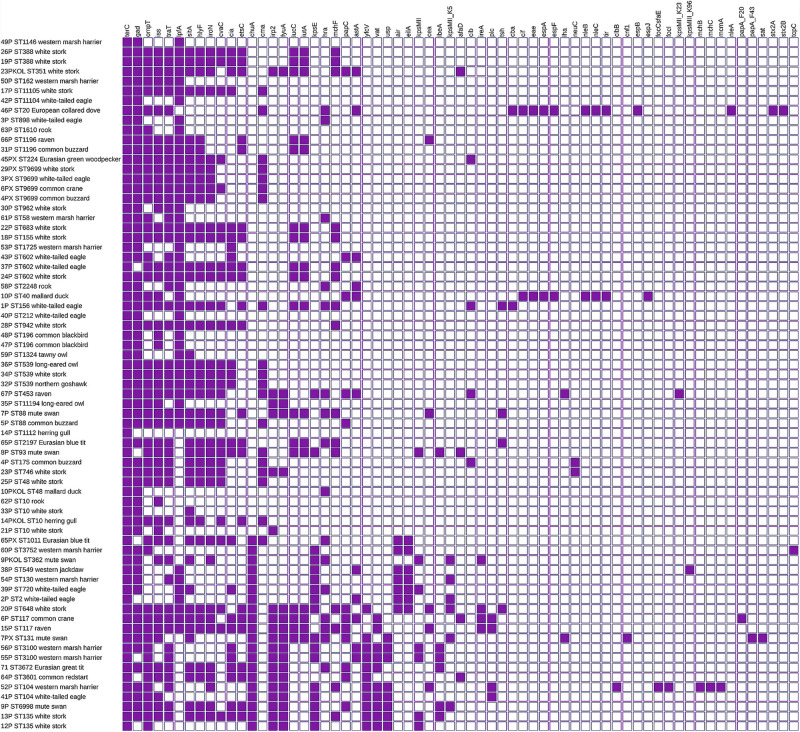
Virulence of *E. coli* isolated from wild birds. Virulence factor by sequence type and bird’s species. Full and empty squares mean the presence or absence of virulence factors. The matrix was visualized with iTOL v5.

Virulence factors were noted in all tested strains. One *E. coli* possessed a single virulence gene; the remaining carried at least three virulence genes. The majority of *E. coli* (*n* = 47) contained 10 or more virulence factors. In nine strains, 20 up to 26 virulence genes were found simultaneously. A huge diversity (*D* = 0.959) of virulence factors amounting to 65 different determinants was noted. The most prevalent genes were *terC* (*n* = 71), *gad* (*n* = 63), *ompT* (*n* = 51), *iss* (*n* = 49), *traT* (*n* = 48), *lpfA* (*n* = 46), and *sitA* (*n* = 42) (Figure 2). One of the *E. coli* isolated from deceased collared dove (*Streptopelia decaocto*) was recognized as Shiga toxin-producing *E. coli* (STEC). It carried *stx2A* and *stx2B* encoding Shiga toxin and *nleA*–*C* determining non-LEE encoded effectors.

### Phylogenetic Diversity

Multilocus sequence typing (MLST) revealed 52 STs among 71 tested *E. coli* (Simpson diversity index *D* = 0.989). The most abundant were ST10 and ST9699 (four strains each), followed by ST602 and ST539 (three *E. coli* each). Nine ST types (including avian pathogenic ST117) were represented by two strains. One strain belonged to pandemic *E. coli* lineage ST131. The strain was isolated from deceased mute swan, and it was MDR and harbored the CTX-M-15 enzyme. All strains derived from the same sample but different culture media belonged to different STs. The minimum SNP dissimilarities were observed between strains belonging to ST388 (four SNPs). The highest difference of 46,533 SNPs was noted between strain 55P (ST3100) and 54P (ST130). Three novel STs were noted and assigned by EnteroBase as ST11104, ST11105, and ST11194. Those three STs were characterized by new *gy*r alleles (1,037, 1,038, and 1,047, respectively). All *E. coli* belonging to ST9699 were cephalosporin-resistant and carried the *bla*_CMY–2_ gene. One ST10 strain from deceased herring gull was MDR and possessed *inter alia mcr-1.1* and *bla*_TEM–1B_. The remaining three ST10 strains were susceptible (Figure 1).

## Discussion

The impact of resistant bacteria on health and environmental issues is unquestionable. Thus, the identification of potential hazards associated with wildlife becomes critical to recognize environmental reservoirs of AMR and to take appropriate and prompt actions against possible emerging AMR mechanisms (Davies and Davies, 2010). Our study, although based on a convenience sampling of a limited fraction of free-living bird populations, confirms its role in AMR transmission and reveals the zoonotic potential of *E. coli* coming from the avian reservoir.

### AMR in Free-Living Birds—the Possible Impact of Human Activity

The main concerns arising from our study relate to resistance toward antimicrobial classes of the highest priority in human medicine, e.g., cephalosporins, quinolones, polymyxins, and aminoglycosides (World Health Organization [WHO], 2019; European Medicines Agency, 2020), as well as fosfomycin, assigned to substances that should be limited for human prescription in the EU (European Medicines Agency, 2020).

The AMR profiles noted in *E. coli* of avian origin were dominated by resistance toward classes of antimicrobials often used in human and veterinary medicine (Food and Agriculture Organization of the United Nations, 2016; World Health Organization [WHO], 2018; European Medicines Agency and European Surveillance of Veterinary Antimicrobial Consumption, 2019). This finding indicates that ubiquitous birds might be exposed to the anthropogenic impact and acquisition of resistant bacteria through contact with the waste of human or livestock origin. Resistance toward antimicrobial classes that we found prevailing (beta-lactams, tetracyclines, quinolones, aminoglycosides, folate path inhibitors) was also reported in bird communities from distinct geographical areas worldwide, from the Americas through Africa, Europe, Asia to Australia (Nascimento et al., 2003; Guenther et al., 2010b; Literak et al., 2010; Poirel et al., 2012; Veldman et al., 2013; Mohsin et al., 2016; Guenther et al., 2017; Ahlstrom et al., 2018; Marcelino et al., 2019; Zurfluh et al., 2019; Cao et al., 2020; Fuentes-Castillo et al., 2020; Nabil et al., 2020). Moreover, AMR toward those substances was commonly found in *E. coli* from farm animals and reported from other wildlife species (Navarro-Gonzalez et al., 2013; Wasyl et al., 2013, 2018; Ceccarelli et al., 2020). The AMR status of bacteria isolated from birds living in pristine environments of Antarctica—gentoo penguin (*Pygoscelis papua*)—seems to be contrasting. Some studies showed an almost complete lack of resistance in bacteria from penguins, while other studies revealed resistance but simultaneously indicated that the result might have been influenced by human activity (Bonnedahl et al., 2008; Miller et al., 2009; Rabbia et al., 2016; Marcelino et al., 2019).

The direct anthropogenic impact could have caused resistance noted in *E. coli* from birds originating from rescue centers. Although the animals were not treated with antimicrobials, they were fed by humans and kept in human proximity. The inclusion of that group of birds might be considered as a weakness of the study. Nevertheless, we believe that the results may highlight the immediate risk for people dealing with birds. Furthermore, it reveals the potential “microbial pollution” of the environment after the release of birds into their natural habitats.

Exposure to environmental pollution and anthropogenic factors, e.g., human waste, sewage treatment plant effluents, and manure, seems to be the significant cause inducing resistance in migratory species and waterbirds, e.g., white stork, crane, mute swan, and mallard (*Anas platyrhynchos*), that we found. That scenario has also been postulated by other researchers (Cole et al., 2005; Guenther et al., 2012; Bouaziz et al., 2018; Marcelino et al., 2019).

We assume that limited contact with human-related external factors and the environment explains the lowest number of NWT strains observed among birds sampled in nature as a significant part of that avian category constituted predominantly by young birds. Similar conclusions were derived by researchers from Switzerland that also suggested a higher resistance *E. coli* rate in adult birds and the parental transmission of AMR as the most probable in the case of juveniles (Zurfluh et al., 2019).

Deliberating the possible source of AMR in avian species, the position of birds within the trophic interactions should also be taken into consideration. It cannot be excluded that the multidrug resistance found in our study among *E. coli* from raptorial birds might be the aftermath of transmission and accumulation of resistance determinants from their potential prey (Marrow et al., 2009).

### Selected Resistance Mechanisms and Potential Transfer

It should be emphasized that no resistance determinants toward carbapenems were noted among the tested strains, and so far, carbapenem resistance has rarely been reported in Poland in farm animals and other wildlife species (Lalak et al., 2016; Wasyl et al., 2018; Skarżyńska et al., 2020a,b). However, since the first report of carbapenemase-producing *Salmonella* Corvallis from the migratory bird—black kite (*Milvus migrans*) in Germany (Fischer et al., 2013), several new studies that presented carbapenem resistance in bacteria from free-living birds were published (Vittecoq et al., 2017; Bouaziz et al., 2018). One of them described a high prevalence of *bla*_IMP_-producing enterobacteria in the silver gull (*Chroicocephalus novaehollandiae*) on Five Islands of Australia (Dolejska et al., 2016). The authors suggested that colonization of birds with resistant bacteria was a result of the feeding habits of the birds at a local waste depot contaminated with clinical material.

Numerous studies reported the occurrence of ESBLs in free-living birds (Guenther et al., 2012, 2017; Mohsin et al., 2017; Zurfluh et al., 2019). It is worth emphasizing that we found CTX-M-15, ESBL of public health concern, in *E. coli* from a deceased mute swan. Although ESBL genes are frequently described on IncF and IncI plasmids (Rozwandowicz et al., 2018) in case of ESBL-producing strains in our study, the resistance genes were not located on the same contigs as plasmid replicons. The gene *bla*_CTX–M–15_, as well as the other AMR genes in this strain, was located in the chromosome. It has been suggested that the incorporation of AMR genes into the chromosome favors the maintenance of resistance in the bacterial population of *E. coli* (Rodríguez et al., 2014). The occurrence of chromosomally encoded *bla*_CTX–M_ genes was previously reported in studies on avian *E. coli* from Pakistan and Mongolia (Guenther et al., 2017; Mohsin et al., 2017). Moreover, chromosomal integration of *bla*_CTX–M–15_ was described in clinical *E. coli* isolates belonging to the clonal group ST131, and our strain represented this lineage (Rodríguez et al., 2014).

The ESBL encoded by *bla*_SHV–12_ in archival *E. coli* from woodpecker was one of the most prevalent ESBLs associated with nosocomial infections before the increase of CTX-M enzymes (Coque et al., 2008). The SHV-12 was previously reported in *E. coli* from waterbirds in Poland (Literak et al., 2010), but it was found in free-living birds in Spain and The Netherlands as well (Veldman et al., 2013; Oteo et al., 2018). Moreover, spreading of *bla*_SHV–12_ was formerly revealed among indicator *E*. *coli* isolated from food animals in Poland (Lalak et al., 2016).

It should be emphasized that in our study cephalosporin resistance was mostly linked to AmpC-type cephalosporinase encoded by *bla*_CMY–2_. The finding of the gene in *E. coli* from blue tit, often found in close vicinity to human settlements, is a cause for concern. During winter, tits are often fed with pork fat and this raises a question on the direction of the gene transmission. It was formerly revealed that in Poland, *bla*_CMY–2_ disseminated among *E. coli* from food-producing animals, e.g., pigs, broilers, and turkeys, as well as from wildlife, e.g., wild boars (Lalak et al., 2016; Wasyl et al., 2018). Among free-living birds, the gene was reported in *E. coli* from species associated with aquatic environments and birds of prey (Poirel et al., 2012; Veldman et al., 2013; Ahlstrom et al., 2018).

AMR mechanisms toward quinolones in tested *E. coli* were dominated with mutations in quinolone resistance determining region (QRDR), and this result might indicate the presence of quinolone selection pressure in the environment. Naturally, drug residues in the environment are subjected to photo- and biodegradation, but the latter process seems to have lower rates for quinolones as synthetic compounds (Martinez, 2009). It was formerly revealed that even at very low concentrations quinolones might select for resistance (Gullberg et al., 2011; Andersson and Hughes, 2012). Our results show that QRDR mutations seem to spread clonally in certain lineages of *E. coli*. QRDR mutations were reported previously in *E. coli* isolated from gulls and birds of prey. Furthermore, it was demonstrated that the same *E. coli* lineages were present in wastewaters, streams, and gulls (Vredenburg et al., 2014). Former research on *E. coli* derived from free-living birds with septicemia also indicated the role of QRDR mutations but reported only mutation in gyrase A subunit (*gyrA*) leading to the Ser-83Leu amino acid substitution (Jimenez Gomez et al., 2004). Single mutations in *parC* and *parE* noted here did not affect the quinolone susceptibility of the strains (Heisig, 1996; Vila et al., 1996; Komp Lindgren et al., 2003; Ling et al., 2003).

In our study, only a few *qnr* genes were noted conversely to a previous report on *E. coli* from free-living birds sampled on the Polish Baltic sea coast, which revealed *qnrS* gene presence in quinolone-resistant strains accompanied by *gyr* and *par* mutations in some of them (Literak et al., 2010). It should be underlined that the majority of former studies on quinolone resistance in free-living birds from the United States, Europe, and Asia were focused mostly on plasmid-mediated resistance mechanisms and reported the occurrence of *qnrS*, *qnrB*, and *aac(6′)-Ib-cr* (Literak et al., 2010, 2012; Halova et al., 2014; Oh et al., 2016; Mohsin et al., 2017). Interestingly, our study confirmed the presence of *aac(6′)-Ib-cr* that determines resistance toward quinolones and aminoglycosides along with beta-lactamases encoded by *bla*_CTX–M–15_ and *bla*_OXA–1_ in *E. coli* from a deceased mute swan, supporting the theory on the spread of these resistance gene sets among bird communities (Literak et al., 2010; Veldman et al., 2013; Vredenburg et al., 2014).

The cause for concern was finding plasmid-mediated colistin resistance (*mcr-1*) in deceased mute swan and herring gull. It is worth noting that the first cases of the *mcr-1* occurrence in free-living birds were published in 2016 (Mohsin et al., 2016; Ruzauskas and Vaskeviciute, 2016). Similar to our results, these studies reported *mcr-1* in species associated with aquatic environments: herring gull and coot (*Fulica atra*). A recent study from Egypt revealed over 10% prevalence of *mcr-1* in bacteria from resident birds (e.g., pigeons, crows) and even 20% prevalence of the gene in migratory waterfowls birds (Ahmed et al., 2019). Moreover, the gene was detected in water samples collected in the area of bird trapping. All the above results indicate that birds might be considered an important vector of colistin resistance. Previous studies from Poland showed the wide spread of *mcr-1* among food-producing animals, particularly turkeys (Zaja̧c et al., 2019). Corresponding to our study, the gene was revealed *inter alia* on IncX4 plasmid and occurred mostly in isolates with colistin MIC close to ECOFF (2 mg/L). The mentioned Egyptian study also reported *mcr-2* presence, although less frequently (Ahmed et al., 2019). The gene was found neither in this research nor in previous studies from Poland (Literak et al., 2010; Zaja̧c et al., 2019).

The finding of *fosA7* gene encoding fosfomycin resistance in *E. coli* derived from birds remaining at rescue centers drew our attention. White-tailed eagle colonized with *E. coli* carrying *fosA7* was released into the natural habitat becoming a source of the resistance in the environment. This perfectly illustrates the feasibility of “microbial pollution” by AMR determinants and resistant bacteria. Recently, the *fosA7*-like gene and other fosfomycin resistance gene, namely *fosA3*, were found in Andean condors (*Vultur gryphus*) (Fuentes-Castillo et al., 2020). Earlier, *fosA7* was described in *Salmonella* isolated from broiler chickens in Canada, as well as from retail meat and clinical incidents in the United States (Rehman et al., 2017; Keefer et al., 2019). The gene was also noted in *E. coli* recovered from soil exposed to anthropogenic activities in North Carolina (Balbin et al., 2020). In Europe, the presence of fosfomycin resistance gene *fosA3* in a *Salmonella* isolated from the migratory bird black kite was reported in Germany (Villa et al., 2015).

Although we noted an infrequent (5.7%) occurrence of resistance to aminoglycosides represented exclusively by gentamicin, we found a spectrum of genes determining resistance toward other compounds of this group, e.g., streptomycin. This result proved that application of phenotypical methods often limited to several antimicrobials, or identification of selected resistance determinants, may lead to underestimation of real AMR status. Similar conclusions were also presented in another study (Rega et al., 2021). That was perfectly illustrated by the *E. coli* recognized as wild type, isolated from a marsh harrier sampled in nature. The analysis revealed that the strain carried aminoglycoside phosphotransferases encoded by *aph(3″)-Ib* and *aph(6)-Id*. On the other hand, identification of the gene (i.e., *mcr-1* discussed earlier) does not always mean resistance. Those findings drive attention to the everlasting discussion of phenotype–genotype congruence and the superiority of the methods applied for testing both aspects.

### Phylogeny and Virulence—A Threat to Public and Animal Health

A variety of sequence types was revealed among the tested *E. coli* including some relevant lineages. Furthermore, a wide range and number of virulence factors were observed.

A single isolate from deceased herring gull belonged to ST10, one of the clinically important clones, and carried *mcr-1*. Such an *E. coli* ST10 with *mcr-1* was reported in the Sultanate of Oman from human bloodstream infection (Mohsin et al., 2018). A similar strain was noted in a clinical case in Uruguay (Papa-Ezdra et al., 2020). That *E. coli* variant was also observed in poultry from Poland and China (Yang et al., 2017; Zaja̧c et al., 2019) and in agricultural soil of Algeria (Touati et al., 2020).

Another highly virulent lineage—ST131 often associated with extended-spectrum β-lactamase CTX-M-15 spread, was reported as predominant among extraintestinal pathogenic *E. coli* (ExPEC) (Coque et al., 2008; Nicolas-Chanoine et al., 2014). In Poland, the ST131 clone was a frequent cause of neonatal infections (Chmielarczyk et al., 2013). Indeed, our ST131 from deceased mute swan strain possessed several virulence determinants specific for ExPEC pathotype such as enabling colonization (*pap*) and adherence (*iha*), as well as determining the outer membrane hemin receptor (*chuA*) and secreted autotransporter toxin (*sat*) (Sarowska et al., 2019).

The identification of STEC belonging to ST20 in deceased collared dove captured our attention. Although pigeons were pointed out as a STEC reservoir, most of the researches indicated the presence of Stx2f toxin in the tested strains. A recent study concerning Stx2f-carrying *E. coli* demonstrated that strains responsible for human infections do not directly originate from the pigeon reservoir (van Hoek et al., 2019). However, the *E. coli* tested here possessed two subtypes of toxin Stx2, including Stx2A, which has previously been described as more potent in causing clinical outcomes (Fuller et al., 2011). Moreover, the study on STEC from Switzerland reported ST20 clone carrying *stx2A* from human patients (Fierz et al., 2017).

We also noted *E. coli* ST117 strains from deceased crane and raven harboring *papC* (outer membrane usher P fimbriae), accompanied with *fyuA* (yersiniabactin receptor), *iucC* (aerobactin synthetase), *iroN* (enterobactin siderophore receptor protein), *vat* (vacuolating autotransporter toxin), and *iss* (increased serum survival). It might be perceived as a poultry health risk since the genes were previously reported in avian pathogenic *E. coli* (APEC) ST117 resulting in increased mortality and colibacillosis in broilers in Nordic countries (Ronco et al., 2017).

As a flash point for the study, the archival MDR *E. coli* woodpecker isolate was assigned to the global clone ST224. Noteworthy, our strain possessed high pathogenicity potential carrying multiple virulence genes. MDR *E. coli* belonging to ST224 lineage were previously isolated from patients with urinary tract infections in China (Cao et al., 2014). MDR strains assigned to ST224 were identified among *E. coli* from retail food (chicken carcasses and ground beef) in Egypt (Ramadan et al., 2020). ST224 *E. coli* were also noted among ESBL-producing strains from food-producing animals and wastewater samples in Tunisia (Sghaier et al., 2019). Moreover, *E. coli* ST224 was reported to cause a fatal pneumonia infection in a domestic cat (*Felis catus*) (Silva et al., 2018).

## Conclusion

All of the above findings indicate free-living bird populations represented by our study group might be considered a source or vector of *E. coli* posing a possible threat to public and animal health. Identification of resistance toward several antimicrobial classes including substances of the highest priority for human medicine, e.g., cephalosporins and quinolones in all tested groups of birds, verified that free-living birds constitute a meaningful AMR reservoir and vector.

Nothing in nature is lost. All pollution of the environment, farmlands, and water might become a possible source of AMR determinants for animals. In consequence, animals affected by resistant bacteria turn into a vector of AMR transmission. Our study shows that the clonal spread of *E. coli* lineages of public and animal health relevance is a serious avian-associated hazard.

## Data Availability Statement

The original contributions presented in the study are publicly available. This data can be found here: the European Nucleotide Archive (ENA), accession number: PRJEB42669, (http://www.ebi.ac.uk/ena/data/view/PRJEB42669).

## Ethics Statement

The animal study was reviewed and approved by the Ministry of the Environment (approval number: DL-III.6713.11.2018.ABR) General Directorate for Environmental Protection (approval number: DZP-WG.6401.03.2.2018.jro) Regional Directorate for Environmental Protection (RDOŚ) in Lublin (approval number: WPN. 6401.6.2018.MPR).

## Author Contributions

MS and DW designed the experiments. MP, JW, and WK performed the sampling campaign. MS and MZ performed the experiments. MS and AB performed the NGS analyses. ŁB performed the statistical analyses. MS prepared the manuscript. All authors discussed the results, reviewed and edited the manuscript, and read and approved the final version of the manuscript.

## Conflict of Interest

The authors declare that the research was conducted in the absence of any commercial or financial relationships that could be construed as a potential conflict of interest.

## Footnotes

1 https://enterobase.warwick.ac.uk/

2 http://www.ebi.ac.uk/ena

3 http://www.ebi.ac.uk/ena/data/view/PRJEB42669
