# Supplementary material for: Antimicrobial Resistance Glides in the Sky—Free-Living Birds as a Reservoir of Resistant Escherichia coli With Zoonotic Potential
Source: Front Microbiol. 2021 Apr 9;12:656223. doi: 10.3389/fmicb.2021.656223 (PMC8062882; doi:10.3389/fmicb.2021.656223)
Supplement: Supplementary file 1 [file Data_Sheet_1.docx]

Supplementary Material

# Supplementary Table 1

| **lp** | **sample no** | **sampling year** | **bird order** | **bird species** | **common name** | **tested group** | **sample type** | **strain no** |
| --- | --- | --- | --- | --- | --- | --- | --- | --- |
| 1 | 1P | 2018 | Accipitriformes | *Haliaeetus albicilla* | white-tailed eagle | deceased | intestines | 1P |
| 2 | 2P | 2018 | Accipitriformes | *Haliaeetus albicilla* | white-tailed eagle | deceased | intestines | 2P |
| 3 | 3P | 2018 | Accipitriformes | *Haliaeetus albicilla* | white-tailed eagle | deceased | intestines | 3P |
|  |  |  |  |  |  |  |  | 3PX * |
| 4 | 4P | 2018 | Accipitriformes | *Buteo buteo* | buzzard | deceased | intestines | 4P |
|  |  |  |  |  |  |  |  | 4PX * |
| 5 | 5P | 2018 | Accipitriformes | *Buteo buteo* | buzzard | deceased | intestines | 5P |
| 6 | 6P | 2018 | Gruiformes | *Grus grus* | crane | deceased | intestines | 6P |
|  |  |  |  |  |  |  |  | 6PX * |
| 7 | 7P | 2018 | Anseriformes | *Cygnus olor* | mute swan | deceased | intestines | 7P |
|  |  |  |  |  |  |  |  | 7PX * |
| 8 | 8P | 2018 | Anseriformes | *Cygnus olor* | mute swan | deceased | intestines | 8P |
| 9 | 9P | 2018 | Anseriformes | *Cygnus olor* | mute swan | deceased | intestines | 9P |
|  |  |  |  |  |  |  |  | 9PKOL ^ |
| 10 | 10P | 2018 | Anseriformes | *Anas platyrhynchos* | mallard | deceased | intestines | 10P |
|  |  |  |  |  |  |  |  | 10PKOL ^ |
| 11 | 11P | 2018 | Anseriformes | *Anas platyrhynchos* | mallard | deceased | intestines | - |
| 12 | 12P | 2018 | Pelecaniformes | *Ciconia ciconia* | white stork | deceased | intestines | 12P |
| 13 | 13P | 2018 | Pelecaniformes | *Ciconia ciconia* | white stork | deceased | intestines | 13P |
| 14 | 14P | 2018 | Charadriiformes | *Larus argentatus* | herring gull | deceased | intestines | 14P |
|  |  |  |  |  |  |  |  | 14PKOL ^ |
| 15 | 15P | 2018 | Passeriformes | *Corvus corax* | raven | deceased | intestines | 15P |
|  |  |  |  |  |  |  |  | 15PKOL # |
| 16 | 3B | 2018 | Accipitriformes | *Haliaeetus albicilla* | white-tailed eagle | deceased | intestines | 39P |
| 17 | 4B | 2018 | Accipitriformes | *Haliaeetus albicilla* | white-tailed eagle | deceased | intestines | 40P |
| 18 | 5B | 2018 | Accipitriformes | *Haliaeetus albicilla* | white-tailed eagle | deceased | intestines | 41P |
| 19 | 7B | 2018 | Accipitriformes | *Haliaeetus albicilla* | white-tailed eagle | deceased | intestines | 42P |
|  |  |  |  |  |  |  |  | 43P ^ |
| 20 | 80B | 2017 | Columbiformes | *Streptopelia decaocto* | collared dove | deceased | intestines | 46P |
| 21 | 66P | 2020 | Passeriformes | *Corvus corax* | raven | deceased | goitre swab | 66P |
| 22 | 67P |  |  |  |  |  | stomach | 67P |
| 23 | 16P | 2018 | Pelecaniformes | *Ciconia ciconia* | white stork | rescue | faeces | - |
| 24 | 17P | 2018 | Pelecaniformes | *Ciconia ciconia* | white stork | rescue | faeces | 17P |
| 25 | 18P | 2018 | Pelecaniformes | *Ciconia ciconia* | white stork | rescue | faeces | 18P |
| 26 | 19P | 2018 | Pelecaniformes | *Ciconia ciconia* | white stork | rescue | faeces | 19P |
| 27 | 20P | 2018 | Pelecaniformes | *Ciconia ciconia* | white stork | rescue | faeces | 20P |
| 28 | 21P | 2018 | Pelecaniformes | *Ciconia ciconia* | white stork | rescue | faeces | 21P |
| 29 | 22P | 2018 | Pelecaniformes | *Ciconia ciconia* | white stork | rescue | faeces | 22P |
| 30 | 23P | 2018 | Pelecaniformes | *Ciconia ciconia* | white stork | rescue | faeces | 23P |
|  |  |  |  |  |  |  |  | 23PCOL ^ |
| 31 | 24P | 2018 | Pelecaniformes | *Ciconia ciconia* | white stork | rescue | faeces | 24P |
| 32 | 25P | 2018 | Pelecaniformes | *Ciconia ciconia* | white stork | rescue | faeces | 25P |
| 33 | 26P | 2018 | Pelecaniformes | *Ciconia ciconia* | white stork | rescue | faeces | 26P |
| 34 | 27P | 2018 | Pelecaniformes | *Ciconia ciconia* | white stork | rescue | faeces | - |
| 35 | 28P | 2018 | Pelecaniformes | *Ciconia ciconia* | white stork | rescue | faeces | 28P |
| 36 | 29P | 2018 | Pelecaniformes | *Ciconia ciconia* | white stork | rescue | faeces | 29P # |
|  |  |  |  |  |  |  |  | 29PX * |
| 37 | 30P | 2018 | Pelecaniformes | *Ciconia ciconia* | white stork | rescue | faeces | 30P |
| 38 | 31P | 2018 | Accipitriformes | *Buteo buteo* | buzzard | released | faeces | 31P |
| 39 | 32P | 2018 | Accipitriformes | *Accipiter gentilis* | northern goshawk | released | faeces | 32P |
| 40 | 33P | 2018 | Pelecaniformes | *Ciconia ciconia* | white stork | released | faeces | 33P |
| 41 | 34P | 2018 | Pelecaniformes | *Ciconia ciconia* | white stork | released | faeces | 34P |
| 42 | 35P | 2018 | Strigiformes | *Asio otus* | long-eared owl | released | faeces | 35P |
| 43 | 36P | 2018 | Strigiformes | *Asio otus* | long-eared owl | released | faeces | 36P |
| 44 | 37P | 2018 | Accipitriformes | *Haliaeetus albicilla* | white-tailed eagle | released | faeces | 37P |
| 45 | 44P | 2018 | Accipitriformes | *Haliaeetus albicilla* | white-tailed eagle | released | faeces | - |
| 46 | 38P | 2018 | Passeriformes | *Corvus monedula* | jackdaw | sampled in nature | faeces | 38P |
| 47 | 47P | 2019 | Passeriformes | *Turdus merula* | blackbird | sampled in nature | faeces | 47P |
| 48 | 48P | 2019 | Passeriformes | *Turdus merula* | blackbird | sampled in nature | faeces | 48P |
| 49 | 49P | 2019 | Accipitriformes | *Circus aeruginosus* | marsh harrier | sampled in nature | faeces | 49P |
| 50 | 50P | 2019 | Accipitriformes | *Circus aeruginosus* | marsh harrier | sampled in nature | faeces | 50P |
| 51 | 51P | 2019 | Accipitriformes | *Circus aeruginosus* | marsh harrier | sampled in nature | faeces | - |
| 52 | 52P | 2019 | Accipitriformes | *Circus aeruginosus* | marsh harrier | sampled in nature | faeces | 52P |
| 53 | 53P | 2019 | Accipitriformes | *Circus aeruginosus* | marsh harrier | sampled in nature | faeces | 53P |
| 54 | 54P | 2019 | Accipitriformes | *Circus aeruginosus* | marsh harrier | sampled in nature | faeces | 54P |
| 55 | 55P | 2019 | Accipitriformes | *Circus aeruginosus* | marsh harrier | sampled in nature | faeces | 55P |
| 56 | 56P | 2019 | Accipitriformes | *Circus aeruginosus* | marsh harrier | sampled in nature | faeces | 56P |
| 57 | 57P | 2019 | Passeriformes | *Turdus philomelos* | song thrush | sampled in nature | goitre swab | - |
| 58 | 58P | 2019 | Passeriformes | *Corvus frugilegus* | rook | sampled in nature | feces | 58P |
|  |  |  |  |  |  |  |  | 58PKOL # |
| 59 | 59P | 2019 | Strigiformes | *Strix aluco* | tawny owl | sampled in nature | feces | 59P |
| 60 | 60P | 2019 | Accipitriformes | *Circus aeruginosus* | marsh harrier | sampled in nature | feces | 60P |
| 61 | 61P | 2019 | Accipitriformes | *Circus aeruginosus* | marsh harrier | sampled in nature | feces | 61P |
| 62 | 62P | 2019 | Passeriformes | *Corvus frugilegus* | rook | sampled in nature | feces | 62P |
| 63 | 63P | 2019 | Passeriformes | *Corvus frugilegus* | rook | sampled in nature | feces | 63P |
| 64 | 64P | 2019 | Passeriformes | *Phoenicurus phoenicurus* | common redstart | sampled in nature | feces | 64P |
| 65 | 65P | 2019 | Passeriformes | *Cyanistes caeruleus* | blue tit | sampled in nature | feces | 65P |
|  |  |  |  |  |  |  |  | 65PX * |
| 66 | 68P | 2019 | Passeriformes | *Larius collurio* | red-backed shrike | sampled in nature | goitre swab | - |
| 67 | 69P | 2019 | Passeriformes | *Turdus philomelos* | song thrush | sampled in nature | goitre swab | - |
| 68 | 70P | 2019 | Passeriformes | *Turdus philomelos* | song thrush | sampled in nature | feces | - |
| 69 | 71P | 2019 | Passeriformes | *Parus major* | great tit | sampled in nature | feces | 71P |
| 70 | 155B | 2013 | Piciformes | *Picus viridis* | green woodpecker | archival | feces | 45PX * |

* strain from MacConkey supplemented with cefotaxime

^ strain from MacConkey supplemented with colistin

# strain excluded from analysis

# Supplementary Table 2

(A) Number of susceptible and resistant strains by bird category

| **bird category** | **susceptible** | **resistant** | N | **% res.** | **95% CI** | |
| --- | --- | --- | --- | --- | --- | --- |
| deceased | 11 | 18 | 29 | 62.1% | 42.3% | 79.3% |
| rescue | 4 | 10 | 14 | 71.4% | 41.9% | 91.6% |
| released | 2 | 5 | 7 | 71.4% | 29.0% | 96.3% |
| sampled in nature | 18 | 2 | 20 | 10.0% | 1.2% | 31.7% |
|  |  |  |  |  |  |  |

N – number of *E. coli* strains

(B) Differences between the bird groups: P-values

| **P-value** | deceased | rescue | released | sampled in nature | correction applied for Chi^2: | | | |
| --- | --- | --- | --- | --- | --- | --- | --- | --- |
| deceased |  |  |  |  |  |  |  |  |
| rescue | 0.7933 |  |  |  | Yates |  |  |  |
| released | 1.0000 | 1.0000 |  |  | Fisher | Fisher |  |  |
| sampled in nature | 0.0003 | 0.0006 | 0.0047 |  | V-square | Fisher | Fisher |  |

Significance level (alpha) after Bonferroni correction for multiple comparisons = 0.00833333

The chi ^ 2 test (with the appropriate correction) showed statistically significant differences between the "birds sampled in nature" group and all other groups (p values in the table above).

**Supplementary Table 3.** Distribution of Minimum Inhibitory Concentration

(A) EUVSEC panel (70 *E. coli* tested)

| **Antimicrobial name and abbreviation** | **NWT** | | **Minimum Inhibitory Concentration value (mg/L)** | | | | | | | | | | | | | | | | | | |
| --- | --- | --- | --- | --- | --- | --- | --- | --- | --- | --- | --- | --- | --- | --- | --- | --- | --- | --- | --- | --- | --- |
|  | n | % | ≤  0.008 | 0.015 | 0.032 | 0.064 | 0.125 | 0.25 | 0.5 | 1 | 2 | 4 | 8 | 16 | 32 | 64 | 128 | 256 | 512 | 1024 | >  1024 |
| Ampicillin (AMP) | 29 | 41.4% |  |  |  |  |  |  | 0 | 5 | 24 | 12 | 0 | 0 | 0 | 29 | 0 |  |  |  |  |
| Ceftazidime (CAZ) | 6 | 8.6% |  |  |  |  |  | 0 | 64 | 0 | 0 | 0 | 6 | 0 |  |  |  |  |  |  |  |
| Cefotaxime (CTX) | 6 | 8.6% |  |  |  |  | 0 | 64 | 0 | 0 | 0 | 6 | 0 |  |  |  |  |  |  |  |  |
| Meropenem (MERO) | 0 | 0.0% |  | 0 | 70 | 0 | 0 | 0 | 0 | 0 | 0 | 0 | 0 | 0 | 0 |  |  |  |  |  |  |
| Gentamicin (GEN) | 4 | 5.7% |  |  |  |  |  | 0 | 46 | 18 | 2 | 0 | 0 | 1 | 3 | 0 |  |  |  |  |  |
| Nalidixic acid (NAL) | 22 | 31.4% |  |  |  |  |  |  |  |  | 0 | 45 | 2 | 1 | 0 | 0 | 22 | 0 |  |  |  |
| Ciprofloxacin (CIP) | 25 | 35.7% | 0 | 42 | 3 | 0 | 0 | 5 | 2 | 1 | 0 | 0 | 17 | 0 |  |  |  |  |  |  |  |
| Sulfamethoxazole (SMX) | 20 | 28.6% |  |  |  |  |  |  |  |  | 0 | 0 | 15 | 29 | 6 | 0 | 0 | 0 | 0 | 20 | 0 |
| Trimethoprim (TMP) | 16 | 22.9% |  |  |  |  | 0 | 52 | 2 | 0 | 0 | 0 | 0 | 0 | 16 | 0 |  |  |  |  |  |
| Colistin (CST) | 0 | 0.0% |  |  |  |  |  |  | 0 | 67 | 3 | 0 | 0 | 0 | 0 | 0 | 0 |  |  |  |  |
| Azitromycin (AZM) |  | NI |  |  |  |  |  |  |  | 0 | 34 | 27 | 9 | 0 | 0 | 0 | 0 |  |  |  |  |
| Chloramphenicol (CHL) | 9 | 12.9% |  |  |  |  |  |  |  |  |  | 0 | 61 | 0 | 4 | 1 | 4 | 0 |  |  |  |
| Tetracycline (TET) | 29 | 41.4% |  |  |  |  |  |  |  | 0 | 41 | 0 | 0 | 0 | 3 | 26 | 0 |  |  |  |  |
| Tigecycline (TGC) | 0 | 0.0% |  |  |  |  | 0 | 69 | 1 | 0 | 0 | 0 | 0 | 0 |  |  |  |  |  |  |  |

(B) EUVSEC2 panel (6 *E. coli* tested)

| **Antimicrobial name and abbreviation** | **NWT** | | **Minimum Inhibitory Concentration value (mg/L)** | | | | | | | | | | | | | | | | | | |
| --- | --- | --- | --- | --- | --- | --- | --- | --- | --- | --- | --- | --- | --- | --- | --- | --- | --- | --- | --- | --- | --- |
|  | n | % | ≤  0.008 | 0.016 | 0.032 | 0.064 | 0.125 | 0.25 | 0.5 | 1 | 2 | 4 | 8 | 16 | 32 | 64 | 128 | 256 | 512 | 1024 | >  1024 |
| Cefotaxime (CTX) | 6 | 100.0% |  |  |  |  | 0 | 0 | 0 | 0 | 0 | 4 | 0 | 1 | 0 | 1 | 0 |  |  |  |  |
| Ceftazidime (CAZ) | 6 | 100.0% |  |  |  |  | 0 | 0 | 0 | 0 | 0 | 0 | 4 | 1 | 1 | 0 | 0 | 0 |  |  |  |
| Cefotaxime/Clavulanic acid (CTX/CLA) |  | NI |  |  | 0 | 1 | 0 | 0 | 0 | 0 | 3 | 1 | 0 | 1 | 0 | 0 | 0 |  |  |  |  |
| Ceftazidime/ Clavulanic acid (CAZ/CLA) |  | NI |  |  |  | 0 | 1 | 0 | 0 | 0 | 0 | 3 | 1 | 0 | 1 | 0 | 0 | 0 |  |  |  |
| Temocillin (TEM) |  | NI |  |  |  |  |  | 0 | 0 | 0 | 0 | 1 | 5 | 0 | 0 | 0 | 0 |  |  |  |  |
| Cefepime (FEP) | 4 | 66.7% |  |  | 0 | 0 | 2 | 2 | 1 | 0 | 0 | 1 | 0 | 0 | 0 | 0 |  |  |  |  |  |
| Cefoxitin (FOX) | 5 | 83.3% |  |  |  |  |  | 0 | 0 | 0 | 0 | 0 | 1 | 0 | 2 | 3 | 0 |  |  |  |  |
| Ertapenem (ETP) | 0 | 0.0% | 0 | 1 | 4 | 1 | 0 | 0 | 0 | 0 | 0 | 0 |  |  |  |  |  |  |  |  |  |
| Imipenem (IPM) | 0 | 0.0% |  |  |  | 0 | 5 | 1 | 0 | 0 | 0 | 0 | 0 | 0 | 0 |  |  |  |  |  |  |
| Meropenem (MEM) | 0 | 0.0% |  | 0 | 6 | 0 | 0 | 0 | 0 | 0 | 0 | 0 | 0 | 0 | 0 |  |  |  |  |  |  |

White zones in MIC values display the applied antimicrobial dilution ranges. Red vertical lines indicate EUCAST epidemiological cutoff values applied as interpretative criteria as defined in Directive 2013/652/EC.

NWT – non-wild type, defines microbiologically resistant isolates with MIC value higher than epidemiological cutoff value; NI – no interpretation criteria available

#
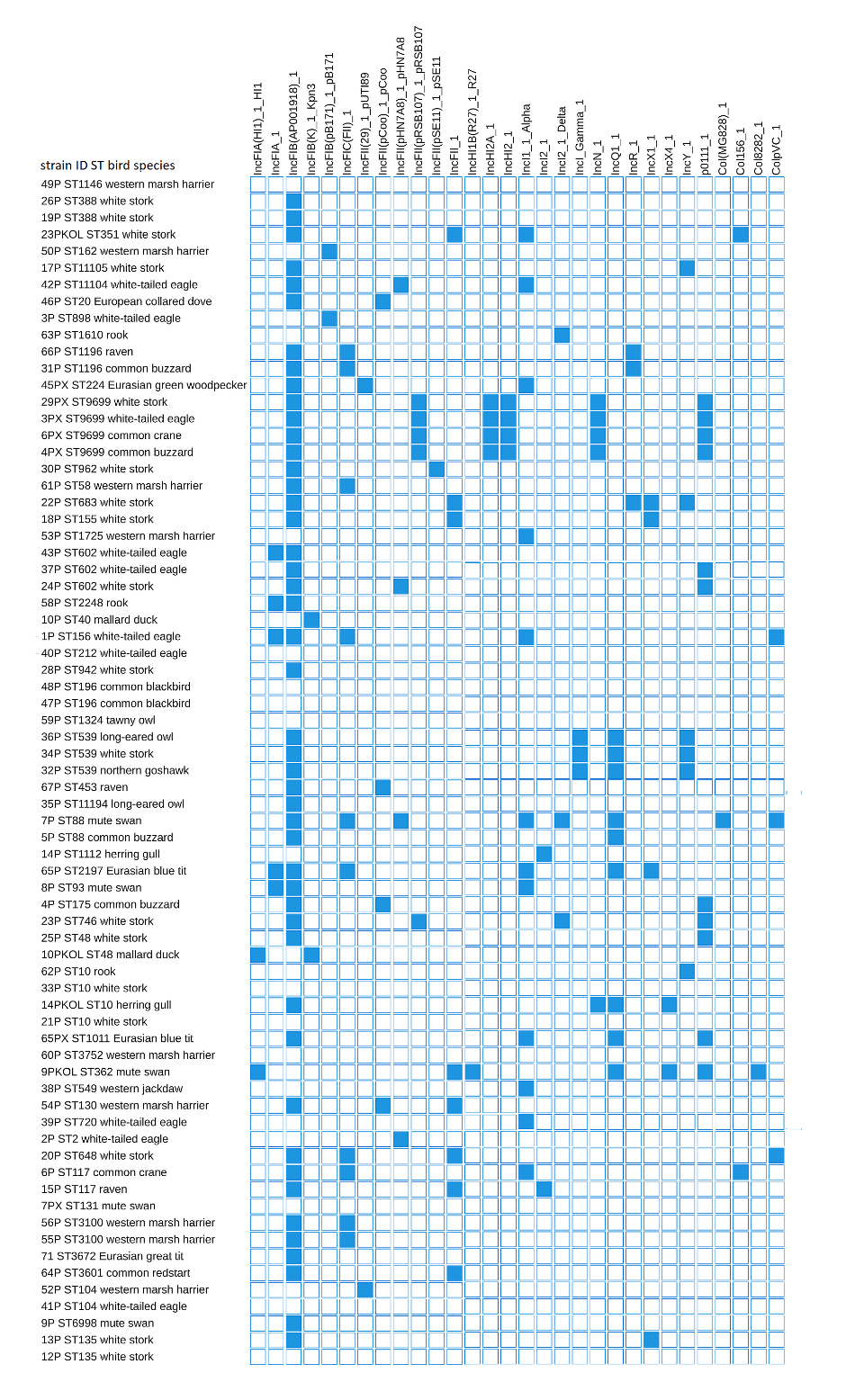
Supplementary Figure 1 Plasmid replicons in tested *E. coli.*

Full and empty squares depicture replicon presence or absence, respectively. An online tool iTOLv5 was applied for the matrix visualization.
